# Unlocking the potential of probiotic administration in caries management: a systematic review

**DOI:** 10.1186/s12903-024-03893-8

**Published:** 2024-02-10

**Authors:** Pedro C. Lopes, Ana T. P. C. Gomes, Karina Mendes, Letícia Blanco, Maria J. Correia

**Affiliations:** 1 Faculty of Dental Medicine, Center for Interdisciplinary Research in Health, Universidade Católica Portuguesa, Viseu, 3504-505 Portugal; 2 Department of Surgery, Universidad de Salamanca, Salamanca, Spain

**Keywords:** Dental caries, Probiotics, Caries risk, Caries management

## Abstract

**Background:**

The use of prebiotics and/or probiotic bacteria with the potential to modulate the oral ecosystem may play an important role in the prevention and management of dental caries. To assess the evidence of the potential of pre/probiotics both in the prevention and treatment of dental caries, we focused on the PICO question “In individuals with caries, after probiotic administration, is there an improvement in outcomes directly related to caries risk and development?“.

**Methods:**

An extensive systematic search was conducted in electronic databases PubMed, Web of Science, Scopus and Cochrane, to identify articles with relevant data. This systematic review included trials performed in Humans; published in English; including the observation of patients with caries, with clear indication of the probiotic used and measuring the outcomes directly involved with the cariogenic process, including the quantification of bacteria with cariogenic potential. To evaluate the methodological quality of the studies, the critical assessment tool from the Joanna Briggs Institute was used.

**Results:**

Eight hundred and fifty articles, potentially relevant, were identified. Following PRISMA guidelines 14 articles were included in this systematic review. Outcomes such as reduction of cariogenic microorganism counts, salivary pH, buffer capacity, and caries activity were assessed. The probiotic most often referred with beneficial results in dental caries outcomes is *Lacticaseibacillus rhamnosus*. Regarding the most used administration vehicle, in studies with positive effects on the caries management, probiotic supplemented milk could be considered the best administration vehicle.

**Conclusions:**

Evidence suggests a beneficial effect of probiotic supplemented milk (*Lacticaseibacillus rhamnosus*) as an adjuvant for caries prevention and management. However, comparable evidence is scarce and better designed and comparable studies are needed.

## Background

Tooth decay is one of the most common chronic infectious diseases and results from a synergistic and complex interaction between bacteria, diet, and susceptible host factors such as teeth and saliva [1]. The development of caries lesions on tooth tissues involves dynamic biological processes, in which acids produced by bacterial fermentation of dietary carbohydrates affects the demineralization of tooth tissues. Repeated acidification leads to the selection of acid-producing and highly acid-resistant organisms. Frequent instances of low pH cause the demineralization-remineralization balance to proceed towards tooth mineral loss. This disturbance of the oral environment is caused by the availability of fermentable carbohydrates in the diet on which acidogenic and acid- tolerant microorganisms thrive upon. Therefore, changes in the composition and biochemical activity of oral biofilms are important determinants of the aetiology of dental caries [2]. Excessive acidification of the oral environment by aciduric species such as Lactobacillus spp. and *Streptococcus mutans* (*S. mutans*) is directly associated with the development of dental caries. However, species with low acid tolerance, such as *Streptococcus salivarius* (*S. salivarius*) and *Streptococcus gordonii* (*S. gordonii*), produce a large amount of alkali, which plays an important role in the acid-base balance of the oral cavity. Tooth demineralization can progress from enamel to dentin but caries progression in the less mineralized dentin is associated a higher pH than what is observed for enamel caries [1]. Therefore, the microbiota of dentin caries differs from that of enamel caries and includes *Lactobacillus, Bifidobacterium, Scardovia, Actinomyces* and *Prevotella* species, capable of acidic and proteolytic activity [3]. The use of prebiotics and/or probiotic bacteria with the potential to modulate the oral ecosystem may play an important role in the prevention and management of dental caries. This topic has attracted the interest of several research groups in the last decades. The World Health Organization defines probiotics as “Live microorganisms which, when administered in adequate amounts, confer a health benefit to the host” [4]. Traditionally, probiotic bacteria mainly *Lactobacillus ssp.*. and *Bifidobacterium ssp.*, have been used in the prevention and treatment of gastrointestinal infections (caused by *Salmonella typhimurium, Clostridium difficile* and *Mycobacterium tuberculosis*) or diseases (such as acute colitis) including caries [5–7]. The mechanism of action underlying probiotic therapy in the oral cavity is based on the hypothesis that harmless bacteria could occupy the niche of pathogenic or opportunistic microorganism in the biofilm [8] However, this effect cannot be generalized, as everyone’s microbiome is unique, and probiotic effects are difficult to predict. It is thought that probiotic bacteria interact not only with the commensal microbiota (excluding or inhibiting pathogens) but also with the host by modulating the immune responses with local and systemic effects [8]. Pathogen exclusion or inhibition occurs both by the production of antimicrobial substances which affect specific community members and through the competition for nutrients or attachment receptors [9]. Nevertheless, the specific mechanisms of action are not clearly identified and understood. In the case of caries, the effect of the probiotic in the oral cavity will result from the interaction of probiotic bacteria with the biofilm, inhibiting and hindering the growth of pathogens through the production of hydrogen peroxide and bacteriocins, and by stimulating the immune response that locally results in increased production of IgA and stimulation of phagocytosis [10]. Another mechanism suggested is that probiotics may prevent cariogenic dental plaque formation directly by adhering to the tooth surface [11] or indirectly by neutralizing free electrons [12].

Probiotics are often associated with prebiotics. The definition of prebiotic has evolved and is currently described as “substrate that is used selectively by host microorganisms conferring a health benefit” [13]. In terms of caries prevention and management, this would include nutrients for microorganisms that inhibit acidogenic and aciduric microbes and/or enhance pH recovery by generating alkali from these nutrients [14]. The two main sources of alkali in the oral cavity are urea and arginine, which, when metabolized by some oral bacteria, result in the production of ammonia and lead to an increase in pH [15]. Thus, prebiotics and probiotics demonstrate the potential to have preventive and therapeutic effects and can be used to prevent or even to treat the disease after it is installed. Probiotics can prevent the oral biofilm from being environmentally “stressed”, enhancing the symbiosis associated with health, as well as “repairing” a dysbiotic biofilm associated with disease [16].

The potential of the use of probiotics in caries prevention has been addressed by some researchers in systematic reviews. Recent reviews considering the impact of probiotics in children have shown that the use of probiotics presented a positive effect in decreasing *S. mutans* counts in saliva [17, 18]. However, Meng and coworkers [18] found that the effect existed when counts of *S. mutans* were done in saliva but not in plaque samples. Meta-analysis have reported that while *S. mutans* counts decreased with the use of probiotics, the same was not observed for *Lactobacilus* counts, neither in saliva nor in plaque [17, 18].

There are also some reviews focusing on a specific probiotic microorganism such as Hao et al. [19], which indicates that use of *Bifidobacterium* based probiotics are neither effective in reducing *S. mutans* nor Lactobacillus counts in saliva or dental plaque, nor in reducing the occurrence of caries in deciduous teeth; or Poorni et al. [20] which analyzed *Streptococcus* strains as probiotics and showed that in vitro promising results do not translate into in vivo clinical benefits. Regardless of the population studied, strains included in the analysis, or the main conclusions drawn from the reviews, authors are unanimous in the need for more data to better support the use of probiotics to promote better oral health.

In the reviews mentioned, the studies in the analyses often included healthy individuals [18, 19, 21, 22], the most recent were focused on children [17, 18, 22, 23], and in some cases, the impact of the probiotic use was not measured as a decrease in cariogenic bacteria [24]. With this review a clear focus on the impact of probiotics on cariogenic bacterial counts in individuals (regardless of age) with previous caries experience is proposed.

Therefore, the objective of this review is to assess the evidence of the potential of probiotics both in the prevention and treatment of dental caries as adjuvant approaches in caries management.

## Methods

This systematic review was conducted following the Preferred Reporting Items for Systematic reviews and Meta-analysis (PRISMA) guidelines and has been recorded in OSF Registries, with the registration DOI: 10.17605/OSF.IO/VF5NG. The focused question was determined according to the Population, Intervention, Comparison and Outcome (PICO) strategy (Table 1).

**Table 1:** Question PICO answered in this study

PICO	
***P*** *opulation*	Individuals with caries
***I*** *ntervention*	Probiotic /Prebiotic administration
***C*** *omparison*	Before and after probiotic / prebiotic administration
***O*** *utcomes*	Improvement of clinical and/or microbiological parameters related to caries risk and development

An extensive systematic literature search was conducted in the electronic databases PubMed, Web of Science, Scopus and Cochrane on November 1st, 2022 covering a 10 year period. The search was conducted to identify articles with relevant data to answer the PICO question “In individuals with caries, after probiotic administration, is there an improvement in outcomes directly related to caries risk and development?” (Table 1). The search was carried out using the following terms and query: (caries OR dental caries OR tooth decay) AND (probiotics OR prebiotics). To narrow the analysis only articles reporting results on the main genera described in the literature as having cariogenic potential such as S*treptococcus*, *Lactobacillus*, *Actinomyces*, *Prevotella* and *Bifidobacterium* [25] were included. The results of the different bases were combined to eliminate duplicated documents and articles were screened by tittle and abstract for eligibility. When the title or abstract did not provide sufficient information regarding the inclusion criteria, full text was obtained and analysed. Two researchers independently participated in all processes (PL and AG), including article selection, data extraction and risk of bias analysis. Disagreement regarding inclusion of specific articles between the reviewers was discussed with a third author (MC). To evaluate methodological quality of the studies, the critical assessment tool - Joanna Briggs Institute (JBI) was used [26]. This systematic review included randomized controlled trials and clinical trials complying with the following inclusion criteria: studies performed in Humans; published in English; including the observation of patients with caries, with clear indication of the probiotic used and measurement of outcomes directly involved with the cariogenic process, including quantification of bacteria with cariogenic potential, such as those referred to in the search key (*Streptococcus, Lactobacillus, Actinomyces*, *Prevotella* and *Scardovia*). Studies that did not meet all the inclusion criteria or in which the population observed had systemic pathologies that could influence the results such as diabetic, immunosuppressed or polymedicated patients, were excluded. In vitro and animal studies were also excluded.

The same reviewers (PL and AG) collected the data independently, in tables structured in Excel spreadsheets with essential information such as: Pubmed ID when available, title, probiotic in study, baseline characteristics of the study population, study design, probiotic dosage, vehicle of administration, cariogenic bacteria quantified, quantification method, outcomes and conclusions. Extracted outcomes related to the improvement of parameters associated with caries risk and development were: salivary counts of *S. mutans* and *Lactobacillus* in probiotic group, increase in salivary pH, appearance or inactivation of caries, oral microbiome composition changes consistent with a healthier microbiota, and salivary concentration levels of antimicrobial peptides.

## Results

**Fig. 1:**
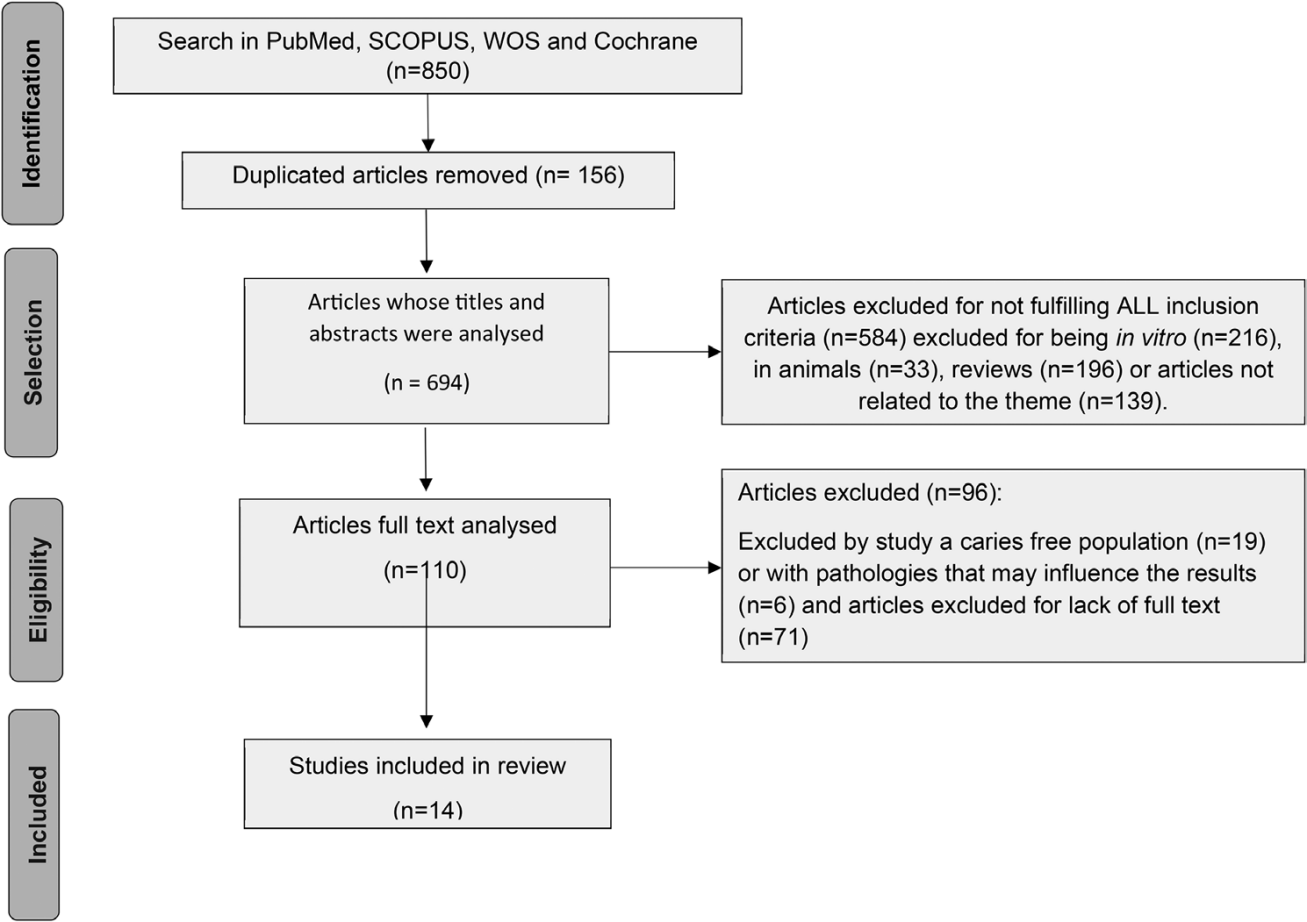
Overview of article selection procedure according to Preferred Reporting Items for Systematic Reviews and Meta-Analyses (PRISMA)

A systematic literature search identified de 850 articles potentially relevant, with 183 publications from PubMed database, 365 from Scopus, 240 from Web of Science and 62 from Cochrane. Duplicated documents were excluded (156), leaving 694 articles in the study. Based on the information provided in the title and abstract, and after article selection and full text analysis, 14 articles were considered in the current review (Fig. 1).

The main reasons for non-inclusion were as follows: 216 were in vitro studies, 139 articles were not related to the topic of probiotics and caries, 33 were animal studies or used bovine enamel on devices used by humans, 196 were reviews and 19 articles were focused on a caries-free population. Six studies addressed diabetics or polymedicated geriatric patients and were therefore excluded. Moreover, 71 articles with no full text available were also excluded. Most of the excluded studies are randomized controlled trial (RCT) protocols registered but without published results.

The studies selected were analyzed regarding the quality of the study according to the JBI criteria and the results of the analysis are presented in Table 2. Almost all aspects of the analysis were fulfilled except for 5 articles [27–31]. It should be noted that in one article [32] there is no real blinding, since the control group is given chewing gum and the experimental group is given yogurt, due to the different” nature” of the treatment, both subjects and researchers knew who was in the control and experimental group. Another article [33] refers to be a double blind, randomized controlled trial, but does not describe in detail how selection, randomization and blinding were performed.

**Table 2:** Results of the analysis of the Joanna Briggs Institute appraisal checklist for critical evaluation of randomized controlled studies

	1. Was true randomization used for assignment of participants to treatment groups?	2. Was allocation to groups concealed?	3. Were treatment groups similar at the baseline?	4. Were participants blind to treatment assignment?	5. Were those delivering the treatment blind to treatment assignment?	6. Were treatment groups treated identically other than the intervention of interest?	7. Were outcome assessors blind to treatment assignment?	8. Were outcomes measured in the same way for treatment groups?	9. Were outcomes measured in a reliable way?	10. Was follow-up complete and, if not, were differences between groups in terms of their follow-up adequately described and analyzed?	11. Were participants analyzed in the groups to which they were randomized?
Campus, 2014	Yes	Yes	Yes	Yes	Yes	Yes	Yes	Yes	Yes	In part	Yes
Rungsri, 2017	Yes	In part	In part	In part	In part	In part	In part	Yes	Yes	Yes	Yes
Villavicencio, 2018	Yes	Yes	In part	Yes	In part	Yes	Yes	Yes	Yes	No	Yes
Zare Javid, 2019	Yes	Yes	Yes	Yes	Yes	Yes	Yes	Yes	Yes	No	Yes
Manmontri, 2019	Yes	Yes	Yes	Yes	Yes	Yes	Yes	Yes	Yes	Yes	Yes
Ferrer, 2019	Yes	Yes	Yes	Yes	Yes	Yes	Yes	Yes	Yes	Yes	Yes
Gedam, 2019	Yes	Yes	Yes	Yes	Yes	Yes	Yes	Yes	Yes	Yes	Yes
Shaalan, 2021	Yes	Yes	Yes	No	No	No	Yes	Yes	Yes	In part	Yes
Wattanarat, 2021	Yes	Yes	Yes	Yes	Yes	Yes	Yes	Yes	Yes	Yes	Yes
Piwat, 2020	Yes	Yes	Yes	Yes	Yes	Yes	Yes	Yes	Yes	Yes	Yes
Ratna Sudha, 2020	Yes	Yes	Yes	Yes	Yes	Yes	Yes	Yes	Yes	No	Yes
*Gandhi, 2020*	Yes	Yes	Yes	Yes	Yes	Yes	Yes	Yes	Yes	No	Yes
Sandoval, 2021	Yes	Yes	Yes	Yes	Yes	Yes	Yes	Yes	Yes	Yes	Yes

A summary of the analysis of the RCTs assessed in Table 2 and a quasi-experimental pilot study [27] are presented in Table 3.

*Lacticaseibacillus* and *Bifidobacterium* are the probiotic most frequently studied, and the most common species are *Lacticaseibacillus rhamnosus* [27, 30, 33–35], *Lacticaseibacillus paracasei* [31, 36, 37] and *Bifidobacterium longum* [27, 30]. Interestingly, two studies had reported the comparison of probiotics (*Bifidobacterium*, *Lacticaseibacillus rhamnosus* and *Lacticaseibacillus plantarum*) with the prebiotics Xylitol and Cinnamon Bark Oil [28, 32].

Most of the studies, reported the consumption of the probiotic as an ingredient of probiotic milk [27, 30, 33, 35, 36, 38, 39], however, the administration via yogurts [31, 32], lozenges and oral tablets [29, 40], adhesive gel or patch [9, 28] and even mouthrinse [34] were found. The daily intake of such probiotics varies between 10^5^ and 10^9^ CFU/mL during the different intervention times.

Most of the studies chose the daily intake of one dose of probiotic [29, 31, 34, 35] whereas Campus et al. opted for a frequency of administration of two doses of probiotic daily. Two other studies reported the intake of one dose of probiotic five times a week [27, 30]. The application of the probiotic bucco-adhesive gel [9] or mucoadhesive patch [28] were administered every 48 h and two times per day, respectively. In 4 studies probiotic intake regimens were compared: one group took one dose daily and the other groups three doses per week [32, 36–38].

**Table 3:** Primary characteristics of the 14 studies included in this systematic review

Entry	Study	Study Desing	Participant number	Participant age (years)	Probiotic/Prebiotic	Administration vehicle	Frequency of administration	Intervention time
1	Campus et al., 2014	RCT	181	6–8	*Lactobacillus brevi*s CD2	Lozenges	Two doses daily	6 weeks of use + 2 weeks follow-up
2	Rungsri et al., 2017	RCT	41	20–25	*Lacticaseibacillus rhamnosus* SD11	Milk	One dose daily	4 weeks of use + 8 weeks follow-up
3	Villavicencio et al., 2018	RCT	363	3–4	*Lacticaseibacillus rhamnosus, Bifidobacterium longum*	Milk	One dose 5 times per week	9 months
4	Angarita-Díaz et al., 2019	Quasi-experimental pilot study	63	3–5	*Lacticaseibacillus rhamnosus, Bifidobacterium longum*	Milk	One dose 5 times per week	3 months
5	Zare Javid et al., 2019	RCT	66	18–30	*Bifidobacterium lactis* Bb12	Yogurt	One dose daily	2 weeks
6	Manmontri et al., 2019	RCT	286	1–5	*Lactobacillus paracasei* SD1	Milk	One dose daily or three times per week	6 months of use + 6 months follow-up
7	Ferrer et al., 2019	RCT	*59*	18–65	*Streptococcus dentisani*	Bucco-adhesive gel	Every 48 h	4 weeks of used + 2 weeks follow-up
8	Gedam et al., 2019	RCT	51	8–12	*Lacticaseibacillus rhamnosus, Lactobacillus acidophilus, Bifidobacterium longum, Saccharomyces boulardii*	Mouth rinse	One dose daily	2 weeks of use + 4 weeks follow-up
9	Shaalan et al., 2021	RCT	96	> 65	Bifidobacterium, Xylitol	Yogurt/chewing gum	One dose daily (for Yogurt) or three doses daily (for chewing gum)	3 months
10	Wattanarat et al., 2021	RCT	286	1–5	*Lactobacillus paracasei* SD1	Milk	One dose daily or 3 times per week	6 months of use + 6 months follow-up
11	Piwat et al., 2020	RCT	469	1–5	*Lactobacillus paracasei* SD1	Milk	One dose daily or 3 times per week	6 months of use + 6 months follow-up
12	Ratna Sudna et al., 2020	RCT	48	5–15	*Bacillus coagulans* Unique IS2	Oral tablets	One dose daily	2 weeks
13	Gandhi et al., 2020	RCT	60	7–10	Cinnamon Bark Oil, *Lacticaseibacillus rhamnosus*, *Lactobacillus plantarum*	Mucoadhesive pacth	Two times per day	2 weeks
14	Sandovalet et al., 2021	RCT	42	2–3	*Lactobobacillus rhamnosus* SP1	Milk	One dose daily	10 months

Regarding intervention time, the discrepancy between studies is notorious. Studies with lower intervention times reported the use of probiotics for 2 weeks [29, 31, 35] or 2 weeks of probiotic usage, followed by an evaluation of the microbial counts or clinical signs after a period of 4 weeks without probiotic use [34] to assess if the changes introduced by the probiotic are maintained.

However, more robust studies with 6 months of probiotic usage and an additional 6 months before follow-up [36, 38–40] or even 9 and 10 months of probiotic usage [30, 35] are the most common design.

The impact of probiotic administration on dental caries outcomes was evaluated in a range of different cohorts, such as children, adolescents, and adults, all in good general systemic health. The studied population has different previous caries experience: from children with at least 1 decay [27] to individuals with 3–10 carious active lesions, including white spot lesions and non-cavitated lesions on enamel surface [9]. Caries identification and evaluation methods were mainly the International Caries Detection and Assessment System (ICDAS II), however other clinical evaluation criteria were also considered, such as plaque and gingival indexes.

In Table 4 the parameters evaluated, and the respective outcomes achieved in the studies selected are summarized.

**Table 4:** Evaluated parameters and the respective outcomes achieved in the 14 studies included in this systematic review

Entry	Study	Clinical Examination	Microbiological analysis	pH evaluation	Oral microbiome composition	Other parameters	Main findings
1	Campus et al., 2014	Reduction of bleeding on probing	Reduction on salivary *S. mutans* concentration	Reduction of plaque pH	N/A	N/A	*L. brevis* CD2 lozenges were effective in reducing important oral health variables.
2	Rungsri et al., 2017	No effects in DMFT, GI, and PI	Reduction on total bacteria and *S. mutans* concentration and increase of *Lactobacilli*. Persistence of *L. rhamnosus* SD11 in test group	No effects on pH	N/A	N/A	Daily consumption of fermented milk containing *L. rhamnosus* SD11 for 4 wk may have beneficial effects on oral health.
3	Villavicencio et al., 2018	No significant differences were attained	*Lactobacillus spp.*concentration reduction	Increase of saliva buffer capacity	N/A	N/A	Daily milk intake supplemented with *L. rhamnosus* and *B. longum* reduces the *Lactobacillus spp*. Counts and increases the saliva buffer capacity in preschool children
4	Angarita-Díaz et al., 2019	Positive effect on carious lesions Remineralization	No significant effect on *S. mutans* concentration	No significant effect on pH variation	N/A	N/A	Clinical studies should continue to determine the functional foods effect of supplemented with probiotics with low acid production capacity and define ideal functional foods that promote children oral health.
5	Zare Javid et al., 2019	N/A	*S. mutans* and *Lactobacillus spp.*concentration reduction	N/A	N/A	N/A	Consumption of probiotic yogurt with *B. lactis* Bb12 may modify the oral biofilm
6	Manmontri et al., 2019	N/A	Reduction on salivary and plaque *S. mutans* concentrations. Increase of *Lactobacilli spp.* In saliva and plaque.	N/A	N/A	N/A	Daily or triweekly consumption of milk supplemented with *L. paracasei* SD1 may help prevent preschool children dental caries.
7	Ferrer et al., 2019	Decrease in plate index, gingival index. Increase salivary flow	Efficient colonization of *S. dentisani*	No significant effect on pH variation	Beneficial shift in bacterial composition, with a reduction of several cariogenic organisms.	Increase of salivary calcium and ammonium	The application of *S. dentisani* 7746 improved several clinical and microbiological parameters associated with oral health, supporting its use as probiotic to prevent tooth decay
8	Gedam et al., 2019	N/A	No significant differences in *S. mutans* concentration between groups	N/A	N/A	N/A	Probiotic mouthrinse was equally efficacious in CHX and NaF mouthrinses against *S. mutans*.
9	Shaalan et al., 2021	N/A	Decrease on *S. mutans* concentration between groups	N/A	N/A	N/A	Probiotic yogurt can be used as an alternative to xylitol in enhancing the oral condition and prevention from caries of geriatric patients
10	Wattanarat et al., 2021	No significant differences were attained	Decrease in *S. mutans* concentration between groups. Increase of *Lactobacillus spp.* Concentration.	N/A	N/A	Elevated salivary HNP1-3 levels in children with early childhood caries upon probiotic supplementation	In the severe-ECC status, consumption of *L. paracase*i significantly enhanced salivary HNP1-3 levels and but reduce *S. mutans* levels, resulting in reduction of caries progression
11	Piwat et al., 2020	Transitions of active caries to inactive caries and decrease of caries risk in probiotic group	N/A	N/A	N/A	N/A	Probiotic milk consumption can modestly prevent new caries, but considerably transform active caries to inactive lesions
12	Ratna Sudna et al., 2020	N/A	Reduction on salivary and plaque *S. mutans* and *Lactobacilli spp.* Concentrations	No significant effect on pH variation	N/A	N/A	14-day administration chewable tablets with probiotic *B. coagulans* Unique IS2 can reduce cariogenic bacteria
13	Gandhi et al., 2020	N/A	Decrease in *S. mutans* concentration.	N/A	N/A	N/A	Cinnamon bark oil incorporated mucoadhesive patch is comparable to the probiotic incorporated patch due to its similarity in the reduction of salivary S. mutans counts.
14	Sandovalet et al., 2021	Increase in the number of teeth with carious lesions in the control group	N/A	N/A	N/A	Increase of salivary hβD-3 in probiotic group	Regular intake of probiotic-supplemented milk in preschool children with high caries risk decreased the occurrence of caries and the salivary levels of hβD-3

Several clinical parameters related to oral health were evaluated and compared between control and probiotic groups in some studies, these include plaque and gingival indexes, salivary flow and bleeding on probing. Among the studies where statistical differences between groups were observed, the increase of salivary flow [9] and in plaque and gingival indexes [9] as well as the reduction of bleeding on probing [9, 40] were the results achieved. In some cases, authors went one step further by evaluating clinical parameters strictly related to caries management. These studies showed that the administration of probiotics caused positive effects on carious lesion demineralization or remineralization [27] in the transition of active to inactive caries, and in the decrease of caries risk [36]. Moreover, one study reported the increase of the number of caries lesion in the control group [35].

Several studies analyzed the concentration of cariogenic *S. mutans* by culture methods. Most reported the decrease of salivary and/or plaque *S. mutans* counts in the probiotic group after the intervention period ( [28, 29, 31–33, 38–40]. Only 2 studies did not observe statistical differences between these groups [27, 34]. Some authors have compared the levels of *Lactobacillus spp*. between groups. The increase of *Lactobacillus spp*. was observed in 3 studies [33, 37, 38] however, the same number of studies reported a decrease in these bacterial genera [29–31]. Interestingly, both *Lacticaseibacillus rhamnosus* [33] and *Streptococcus dentisani (S. dentisani)* [9], were still observed in samples collected in the follow up period. Moreover, when oral microbiome of the individual of each group was analyzed by sequency strategies, a beneficial shift in bacterial composition, with a reduction of several cariogenic organisms was attained in the probiotic group [9].

Salivary and plaque pH were also evaluated by some authors. Only one study reported the decrease in the plaque pH in the probiotic group [40], and 4 studies observed an increase in pH after the probiotic intervention [9, 27, 29, 33]. One study also reported the increase of buffer capacity in the probiotic group [30].

At least, two studies had evaluated the levels of antimicrobial peptides defensins that provide the first line of host defence against a broad spectrum of microorganisms, HNP1-3 and hβD-1 [35, 39]. In both cases, an increase of these defensins levels was achieved in the probiotic group.

## Discussion

Oral microorganisms form complex and dynamic communities that play a crucial role in maintaining oral health. This eubiotic equilibrium may be lost when interaction between microbes and the host is significantly altered, leading to a dysbiotic state which underlies oral and systemic diseases [41].

Dental caries is caused by cariogenic microorganisms (such as *S. mutans* and *Lactobacillus* spp.) in plaque biofilms that can ferment dietary carbohydrates producing acids, lowering pH and leading to mineral loss from the hard tissue of teeth, resulting in cavities [42]. Thus, maintaining a healthy oral microbiome is essential to prevent dental caries. The balanced and diverse oral microbiome can help prevent the overgrowth of cariogenic bacteria and the maintenance of a neutral pH in the mouth. Several factors including diet, hygiene practices, genetics, and environmental factors can, in fact, influence the composition and diversity of the oral microbiome [41, 42].

Regular brushing, along with a balanced and healthy diet and antimicrobial measures with fluoride exposure, can help maintain a healthy oral microbiome and prevent the development of caries [43]. However, the toxicity associated with excess of fluoride or other antibacterial drugs, may cause microecological damage, resulting in re-colonization with secondary opportunistic pathogen and other pathological consequences. In this context, safer and more efficient methods are needed to effectively prevent caries without significant adverse effects [44].

Probiotics are well-known in health promotion and have been extensively studied [45]. In oral health promotion, oral probiotics should be able to adhere and colonize oral tissue including hard non-shedding surfaces and become a part of the biofilm. Moreover, oral probiotics should not be able to perform sugar fermentation, avoiding pH decrease and therefore caries development [46]. Several studies have reported the potential of probiotic use in caries management and development, however it is not easy to find comparable data to support the generalized use of probiotics as adjuvants for treatment and/or prevention of caries. As stated before, there is no lack of studies in the literature, and according to PubMed, in the last 5 years, 8 systematic reviews were published focusing on the study of probiotics’ use in oral health management, namely in caries prevention and/or treatment of preschool children with or without caries [17–24].

However, reviews with results for other age groups (not exclusively children), and which focus on individuals with a previous caries experience are not available. In this review this was the goal. The main finding is that comparison between studies is difficult due to heterogeneity in study design, outcomes measured and procedures/methods to assess caries status and microbial and/or biochemical changes. Although most studies refer a significant positive effect of probiotics administration in caries prevention, more scientific evidence is needed to support these findings. To be able to make more accurate comparisons, in this review, a single study design (RCT) was used.

The results show that the probiotic most often referred as having beneficial results in dental caries outcomes is *Lacticaseibacillus rhamnosus* [30, 33, 35], being the most recommended to be included in clinical studies related to oral health. *L. rhamnosus* has been the object of several studies for its application as a powerful probiotic for human health [47, 48]. This fact is due to its capacity to endure to stressful environments, such low pH, to adhere or compete for colonization in the oral cavity, modulating the innate and adaptive immune responses [33]. Moreover, *Lacticaseibacillus rhamnosus* strains are capable of secreting antimicrobial substances that can inhibit other bacteria strains and can be incorporated into varied delivery food vehicles.

A species which has only recently been considered as a potential oral probiotic is *S. dentisani* [9]. This species is significantly more abundant in caries free individuals [49] and its impact on the oral microbiome has been tested “in vitro” [9]. This bacterium seems to modulate the oral microbiome, promoting a beneficial shift in bacterial composition and leading to a reduction of cariogenic organisms, probably by the production of bacteriocins and increasing the pH buffering capacity of saliva through ammonia production [9]. Despite the promising results obtained in vitro and in pilot studies, further randomized clinical trials assessing administration regimens and vehicles are needed to support the use of *S. dentisani* to prevent tooth decay [50].

Regarding the most used administration vehicle, in studies with positive effects on the caries management, probiotic supplemented milk could be considered the best administration vehicle. Milk is a model ‘nutraceutical’, a food that conveys immunological and other health benefits together with the nutritional contribution [51]. Milk constituents are known to modulate the developing microbiota within the infant gastrointestinal tract. Of the functional ingredients present in milk, oligosaccharides are probably the most important since they act as prebiotics [51]. Although most of the studies published are focused on the effect of human milk oligosaccharides (HMOs) present in human milk in the gastrointestinal tract, it is important to highlight that HMOs play a crucial role in supporting the growth of beneficial gut bacteria, possess anti-adhesive effects that reduce the binding of pathogenic bacteria and modulating effects on immunological processes. Even though the diversity of oligosaccharides content of human milk cannot be successfully reproduced on a large scale, the bioactivities of oligosaccharides from bovine and human milk are similar [33, 51, 52] Therefore, it is possible to assume that the synergy between oligosaccharides from milk and the probiotic *L. rhamnosus* could have a beneficial impact on oral microbiota. Moreover, the consumption of probiotic supplemented milk could be an advantage for children, since milk is widely used in this age group, and it could effectively contribute to the prevention and even to stop the progression of dental caries.

The work of Ferrer and others [9] used *s*equencing approaches to evaluate the community changes after probiotic exposure, rather than focusing on the counts of specific bacteria. The use of culture independent techniques and metagenomic approaches might be more suited to reveal overall changes in the community, better reflecting the impact of probiotics in the community.

As far as intervention time, due to the heterogeneity of data, further randomized clinical trials are recommended to provide better understanding on the influence of intervention time on the probiotic effects. Considering the studies included in this systematic review, longer intervention times (up to 6 months) with longer follow-ups could provide more robust results in decreasing caries risk.

The heterogeneity of results reported is also patent in the frequency of probiotic administration. However, this heterogeneity didn´t affect the main findings of the studies. The results show that, independently of the administration frequency, the use of probiotics has a potential to reduce caries risk and cariogenic bacteria content. Even in the studies that opted for the administration of different probiotic doses in two different groups, the results achieved were similar. In fact, it is not easy to find data in scientific literature for optimal probiotic intake frequency, due to the existence of different probiotic organisms and variables that affect probiotic efficacy [53]. Nevertheless, if the focus is *L. rhamnosus*, the results show that the daily intake of a unique dosage of this probiotic produces promising results in caries risk and cariogenic microorganisms decreases. Besides the intervention time and the need for follow-up, more studies that include caries risk assessment and caries status evaluation, while the microbial quantification and identification are assessed, should also be considered, since studies that combine all this information are scarce. Only with better study designs conjugating clinical and microbial parameters, with higher number of participants, with longer intervention times and with follow-up assessments, will it be possible to support the claims that probiotics are an effective preventive tool for caries.

Evidence from this work suggests that probiotic supplemented milk with *L. rhamnosus* could have positive effects in caries prevention. However, comparable evidence is scarce and, for that reason, new studies on the effect of *L. rhamnosus* in caries prevention and management are needed.

## Conclusion

According to this study, the evidence suggests a beneficial and promising effect on dental caries outcomes by the usage of milk supplemented with *Lacticaseibacillus rhamnosus* as an adjuvant approach to clinical intervention and daily oral hygiene routines. Knowledge in this field would benefit from well-designed studies with a systematic assessment of caries, caries risk and microbial quantification and identification, to elicit a systematic comparison between probiotic composition, vehicles, and administration strategy.

## Data Availability

All data generated or analysed during this study are included in this published article.

## Abbreviations

S. mutans: Streptococcus mutans
S. salivarius: Streptococcus salivarius
S. gordonii: Streptococcus gordonii
S. dentisani: Streptococcus dentisani
PRISMA: Preferred Reporting Items for Systematic reviews and Meta-analysis
PICO: Population, Intervention, Comparison and Outcome
JBI: Joanna Briggs Institute
RCT: randomized controlled trial
ICDAS: International Caries Detection and Assessment System
HMOs: human milk oligosaccharides
